# Maternal near miss in a teaching hospital in the Brazilian Midwest: contributions to care

**DOI:** 10.1590/1980-220X-REEUSP-2024-0200en

**Published:** 2025-01-31

**Authors:** Aline Amorim da Silveira, Ana Paula de Assis Sales, Andreia Insabralde de Queiroz Cardoso, Elen Ferraz Teston, Adriane Pires Batiston, Renata Marien Knupp Medeiros

**Affiliations:** 1 Universidade Federal do Mato Grosso do Sul, Hospital Maria Aparecida Pedrossian, Campo Grande, MS, Brazil.; 2 Universidade Federal de Mato Grosso do Sul, Instituto Integrado de Saúde, Campo Grande, MS, Brazil.; 3 Universidade Federal de Rondonópolis, Faculdade de Ciências da Saúde, Rondonópolis, MT, Brazil.

**Keywords:** Near Miss, Healthcare, Maternal Mortality, Morbidity, Pregnancy Complications, Nursing, Potencial Evento Adverso, Mortalidad Materna, Morbilidad, Complicaciones del Embarazo, Enfermería

## Abstract

**Objective:**

To analyze cases of maternal near miss in a teaching hospital.

**Method:**

This is a cross-sectional study with a quantitative approach. The research was performed in a teaching hospital, in Mato Grosso do Sul, from June to December 2021. Data were collected from physical and electronic medical records, laboratory tests, and prenatal records, using a structured instrument. They were subsequently subjected to descriptive and inferential analysis in Epi Info 7.2.2.6.

**Results:**

Fifty-nine medical records were analyzed, 49 presenting potentially life-threatening condition and ten maternal near misses. Coming from inland cities of the state, gestational risk classification, bleeding and management disorders showed a significant association with maternal near miss.

**Conclusion:**

It becomes necessary to plan tracking actions, such as the implementation of a flow for identifying women with potentially life-threatening conditions, and the implementation of specific protocols with preventive key interventions and notification of maternal near miss, with a view to improving care and consequently reducing the risk of serious maternal outcomes.

## RESUMEN

**Objetivo:**

Analizar casos de *near miss* materno en un hospital universitario.

**Método:**

Se trata de un estudio transversal con enfoque cuantitativo. La investigación fue realizada en un hospital universitario, en Mato Grosso do Sul, de junio a diciembre de 2021. Los datos fueron recolectados de registros de salud físicos y electrónicos, pruebas de laboratorio y registros prenatales, mediante un instrumento estructurado. Posteriormente fueron sometidos a análisis descriptivo e inferencial en Epi Info 7.2.2.6.

**Resultados:**

Se analizaron 59 registros de salud, 49 con condición potencialmente mortal y diez near misses maternos. Ser natural del interior del estado, clasificación de riesgo gestacional, trastornos hemorrágicos y de tratamiento mostraron asociaciones significativas con el near miss materno.

**Conclusión:**

Es necesario planificar acciones de detección, como, por ejemplo, implementación de un flujo de identificación de mujeres con condiciones potencialmente mortales, y la implementación de protocolos específicos con intervenciones clave preventivas y notificación de near miss materno, con miras a mejorar la atención y, en consecuencia, reducir el riesgo de un resultado materno grave.

## INTRODUCTION

Reducing maternal mortality remains a challenge worldwide. The third target of the Sustainable Development Goals (SDGs), launched in 2015, proposed reducing the global maternal mortality rate to at least 70 per 100,000 live births by the year 2030^(1)^.

However, the maternal mortality trends report for the first five years of the SDG (2016–2020) indicated stagnation in progress towards this goal. In 2020, an estimated 287,000 women died worldwide, a third lower than reported from 2000 to 2015, but the average annual global rate of reduction fell from 2.7% to less than 0.04% in the first five years of the SDGs^(2)^.

In Brazil, between 2017 and 2018, all regions of the country witnessed a drop in the maternal mortality ratio (MMR), with the exception of the Central-West region, which saw a significant increase of 14%, and the state of Mato Grosso do Sul, which saw a 53.7% increase. Among the main causes of obstetric death in Brazil, hypertensive diseases, hemorrhagic diseases, and puerperal infection stand out. In the state of Mato Grosso do Sul, from 2017 to 2019, hypertension alone increased by 21%^(3)^.

Maternal mortality is defined as the death of a pregnant woman within 42 days of pregnancy from any cause related to or aggravated by pregnancy or its management, but not from accidental causes; it is an important public health indicator that reflects not only women’s health status, but the quality of health services^(4,5)^.

Like maternal mortality, severe maternal morbidity is used as an indicator to assess the quality of obstetric care. The WHO currently defines severe maternal morbidity as any health condition attributed to or related to pregnancy and childbirth that has a negative impact on the woman’s well-being^(6)^. Within this theme, being considered a tragic puerperal outcome, like death, emerges the maternal near miss (MNN), defined by the WHO as a woman who almost died, but survived the complications taking place during pregnancy, childbirth, or up to 42 days after the end of pregnancy^(7)^.

Its diagnostic criteria were standardized in 2009 by a specialized WHO working group, which, after analyzing different pre-existing concepts in the literature, listed a set of organic dysfunctions capable of leading a parturient or puerperal woman to death. Twenty-five criteria were listed, divided into three groups: clinical, laboratorial, and managerial. Within this context, a list of potentially life-threatening conditions (PLTCs) that are associated with these dysfunctions was also created, including severe postpartum hemorrhage, severe preeclampsia, eclampsia, sepsis/infection and severe systemic infection^(8)^.

The global decline in the maternal mortality rate, especially in developing countries, has made this indicator a less effective strategy for assessing the quality of obstetric care provided in health institutions, so that the analysis of maternal near miss can provide more robust information in this regard. The incorporation of severe maternal morbidity and maternal near miss is recommended in investigations related to maternal health^(8,9)^.

The diagnostic criteria for maternal near miss assist in monitoring the quality of maternal health care and surveillance of severe maternal morbidity. This is because carrying out an audit based on maternal near miss cases allows us to identify the type of assistance received in serious maternal complications. This data can allow the improvement of care, prevention of serious maternal morbidities and, consequently, death^(10)^. Furthermore, the use of this classification standardizes the methodology for identifying serious cases, the comparison of care for pregnant and postpartum women over time, regardless of the location researched^(7,10)^.

As this is a topic still being consolidated in health services, broad and standardized surveys on MNM are rare. Existing studies demonstrate variation in MNM rates depending on the criterion chosen and the population studied, both in terms of the structure of health services and in social aspects. A study carried out in 2022 demonstrated a prevalence of MNM with a minimum variation of two per thousand live births in Italy and a maximum of 37.9 per thousand live births in Ethiopia^(11)^.

In a published protocol of a new Brazilian study in progress, with an evaluation period covering 2021 and 2023, the aim is to estimate the incidence of maternal near miss with detection of a large number of cases. However, as a limiting factor, the data found cannot be validated in hospitals with less than 2,750 births/year, highlighting the importance of knowing the profile of severe maternal morbidity in different locations with services of different characteristics, such as smaller hospitals^(5)^.

The incidence and profile of cases of severe maternal morbidity may have distinct epidemiological behaviors. However, the difference in data and profiles only improve discussions on the topic and validate the evidence. In this sense, the present study aims to identify and analyze cases of maternal near miss in a teaching hospital in the central-west region of Brazil.

## METHOD

### Design of Study

Cross-sectional study, of quantitative approach, which used the criteria Strengthening the Reporting of Observational Studies in Epidemiology (STROBE) for its construction^(12)^.

### Local

It was carried out in the maternity ward of a reference federal teaching hospital, located in the central-west region of Brazil. The maternity ward assists pregnant and postpartum women with spontaneous demand 24 hours a day and referred via the regulatory system, being a reference in high-risk pregnancies and infectious diseases, with an average of 155 births/month, 35% of which of high risk.

### Population and Selection Criteria

Pregnant and postpartum women up to 42 days postpartum, regardless of age, who presented at least one criterion of potentially life-threatening condition (PLTC) were included in the study. The conditions were: bleeding disorder (placenta previa, placenta accreta/increta/percreta, ectopic pregnancy, postpartum hemorrhage and uterine rupture), hypertensive disorder (severe pre-eclampsia, eclampsia, severe hypertension, hypertensive encephalopathy and HELLP syndrome), other systemic disorders (endometritis, pulmonary edema, respiratory failure, seizures, shock, thrombocytopenia <100,000, and thyrotoxic crisis) and management indicators (blood transfusion, central venous access, hysterectomy, ICU admission, prolonged hospitalization above seven days, non-anesthetic intubation, return to the operating room and surgical intervention).

Women who did not speak Portuguese, were unable to answer or understand the questions and the FICF (Free and Informed Consent Form) and/or FIAF (Free and Informed Assent Form for those under 18 years of age), and whose medical records were not found or whose data were insufficient to meet the research objective were excluded from the study.

### Sample Definition

A sample calculation of near miss cases was performed based on the expected number of eligible women with severe maternal outcome (range) according to total number of births^(6)^.

Considering the WHO prevalence of 3.7% and that the average number of annual births from 2015 to 2019 at the University Hospital Maria Aparecida Pedrossian (HUMAP/UFMS) was 1,800, the calculated sample was 60 women, with an increase of 10% for possible losses, with a 5% margin of error, and a 95% confidence level.

### Data Collection

Data were collected from June to December 2021 with the aid of an instrument developed by the main researcher containing sociodemographic variables, care data, obstetric history, puerperal and neonatal outcomes with emphasis on PLTC and MNM criteria. A pilot test was carried out with subsequent discussion with the other researchers to approve the final version of the instrument. Data from physical and electronic medical records of each patient, exams and prenatal records made up the analysis documents.

Daily visits to the hospital were carried out, alternating between morning, afternoon and evening periods. After checking the hospitalization census of the obstetric center and observing diagnoses consistent with PLTC or MNM, pregnant or postpartum women were selected and invited to participate in the study.

After receiving guidance on the objective of the study and agreeing to participate, and in view of the authorization of the FICF/FIAF, the researchers accessed the paper-based medical records, the prenatal card, and the AGHUX system (Management Application for university hospitals), the data management and processing system of the Ebserh Network (Brazilian hospital services company). After collecting institutional documents, the interview was conducted in the ward or in a reserved room in the sector itself, depending on the woman’s availability and the environment.

All interviews and data were collected only once and in a hospital environment, considering women hospitalized for PLPC and Near miss in clinical monitoring or those with the outcome of childbirth and in the puerperal period, with a clinical condition consistent with participation.

### Data Processing

The data were analyzed in the statistics software Epi Info 7.2.2.6., through association tests between two categorical variables, Fisher’s Exact test, and between more than two variables by the Chi-square test, applying a significance level of 5%.

### Ethical Aspects

The project was approved by the Human Research Ethics Committee, opinion no. 4.700.375 and followed all the precepts of resolution no. 466, of December 12, 2012.

## RESULTS

Over a six-month period, 10 MNM events were observed, with a frequency of 16% of the eligible population. There were 1,062 births during the period studied, representing a prevalence ratio of 9.4 per 1000 live births. Fifty-nine women with PLTC were included in the study, of which 78% were non-white and 59.3% had primary education, and the mean age was 26.44 ± 6.64 years (minimum of 13 and maximum of 41 years). There was a loss due to incomplete data and the impossibility of completing it through an interview. The event *Near miss* showed a significant statistical association with being from inland cities of the state (p = 0.01) as seen in Table 1.

**Table 1:** Description of the sociodemographic variables of the 59 participants and their associations with the presence of near miss–Campo Grande, MS, 2024.

Sociodemographic variables	Total	Near miss		Value of p ^(a)^
	% (n)	Yes (10)	No (49)	
Age range				
≤15	3.4 (2)	0.0 (0)	4.1 (2)	0.41
16 a 34	79.7 (47)	70.0 (7)	81.6 (40)	
≥35	16.9 (10)	30.0 (3)	14.3 (7)	
Race/color				
White	22.0 (13)	20.0 (2)	22.4 (11)	1.00
Non-white	78.0 (46)	80.0 (8)	77.6 (38)	
Level of Education				
Incomplete/complete elementary school	59.3 (19)	60.0 (6)	59.2 (29)	0.66
Finished high school	23.7 (14)	20.0 (2)	24.5 (12)	
Finished higher degree	6.8 (4)	0.0 (0)	8.2 (4)	
Not informed^(b)^	10.2 (6)	20.0 (2)	8.2 (4)	
Municipality of Residence				
Capital	81.4 (48)	50.0 (5)	87.8 (43)	0.01*
inland city	18.6 (11)	50.0 (5)	12.2 (6)	

Note:

^(a)^ Analyses between two variables were performed using Fisher’s exact test, while analyses between more than two variables were performed using the chi-square test.

^(b)^ Not reported = corresponds to data missing from the medical record whose amount was disregarded from the inferential statistical analysis. *Significant association p < 0.05.

Regarding obstetric characteristics, it was found that most women were multiparous and underwent prenatal care equally among pregnant women later identified with or without near miss condition (p = 0.72 and p = 0.32, respectively), with early prenatal care (up to the 12th week of pregnancy) happening among most of them.

The obstetric characteristics are described in Table 2. The risk classification at admission showed a statistically significant association with the near miss condition (p = 0.005).

**Table 2:** Description of the obstetric characteristics of the 59 participants and their associations with the presence of near miss. Campo Grande – MS, 2024.

Obstetric characteristics	Total	Near miss		Value of p^(a)^
	% (n)	Yes (10)	No (49)	
Parity				
Primiparous	33.9 (20)	40.0 (4)	32.7 (16)	0.72
Multiparous	66.1 (39)	60.0 (6)	67.3 (33)	
Underwent Prenatal care				
Yes	96.6 (57)	90.0 (9)	98.0 (48)	0.32
No	3.4 (2)	10.0 (1)	2.0 (1)	
Start of prenatal care				
Early	72.9 (43)	90.0 (9)	69.4 (34)	0.33
Late	15.3 (9)	0.0 (0)	18.4 (9)	
Not informed^(b)^	11.9 (7)	10.0 (1)	12.2 (6)	
Number of prenatal consultations				
<6	30.5 (18)	30.0 (3)	30.6 (15)	1.00
≥6	69.5 (41)	70.0 (7)	69.4 (34)	
Prenatal gestational risk				
Usual	47.5 (28)	40.0 (4)	49.0 (24)	1.29
High	47.5 (28)	40.0 (4)	49.0 (24)	
Not informed^(b)^	5.1 (3)	20.0 (2)	2.0 (1)	
Risk classification during hospitalization				
Green/Yellow	84.7 (50)	50.0 (5)	91.8 (45)	0.005*
Orange/Red	15.3 (9)	50.0 (5)	8.2 (4)	
Pre-existing maternal diseases				
Previews	23.7 (14)	30.0 (3)	22.4 (11)	0.49
Gestational	33.9 (20)	40.0 (4)	32.7 (16)	
No pre-existing conditions	42.4 (25)	30.0 (3)	44.9 (22)	
Type of hospital admission				
Spontaneous demand	45.8 (27)	40.0 (4)	46.9 (23)	0.005*
Referral	30.5 (18)	0.0 (0)	36.7 (18)	
*Vaga zero* (exception resource that allows the immediate referral of patients in emergency situations to a referral hospital, even if there is no bed available)	23.7 (14)	60.0 (6)	16.3 (8)	
Period of hospital admission				
1st or 2nd trimester of pregnancy	13.6 (8)	20.0 (2)	12.2 (6)	0.78
3rd trimester of pregnancy	67.8 (40)	60.0 (6)	69.4 (34)	
Immediate puerperium (1st to 10th day)	18.6 (11)	20.0 (2)	18.4 (9)	

Note:

^(a)^ Analyses between two variables were performed using Fisher’s exact test, while analyses between more than two variables were performed using the chi-square test.

^(b)^ Not reported = correspond to data missing from the medical record whose amount was disregarded from the inferential statistical analysis. *Significant association p < 0.05.

Admission through referral was associated with non-near miss (p = 0.0005). The data are detailed in Table 2.

Regarding obstetric care data, cesarean section was the most commonly used birth route, with greater use of antihypertensive medications, both without association with near miss (p = 0.10 and p = 0.65, respectively), as seen in Table 3.

**Table 3:** Description of the characteristics of the assistance of the 59 participants and their associations with the presence of near miss. Campo Grande – MS, 2021.

Assistance features	Total	Near miss		Value of p^(a)^
	% (n)	Yes (10)	No (49)	
Type of delivery				
Vaginal	20.3 (12)	0.0 (0)	24.5 (12)	0.10
Cesarean section	69.5 (41)	90.0 (9)	65.3 (32)	
Not informed^(b)^	10.2 (6)	10.0 (1)	10.2 (5)	
Medication				
Antihypertensive	59.3 (35)	50.0 (5)	61.2 (30)	0.65
Antihemorrhagic	28.8 (17)	30.0 (3)	28.6 (14)	
Outros	11.9 (7)	20.0 (2)	10.2 (5)	
Local of woman’s admission				
Rooming-in care	52.5 (31)	40.0 (4)	55.1 (27)	0.49
OC/IC^(c)^	47.5 (28)	60.0 (6)	44.9 (22)	

Note:

^(a)^ Analyses between two variables were performed using Fisher’s exact test.

^(b)^ Not reported = correspond to data missing from the medical record whose amount was disregarded from the inferential statistical analysis.

^(c)^ OC/IC = Obstetric center/Intermediate or intensive care.

**Figure 1:**
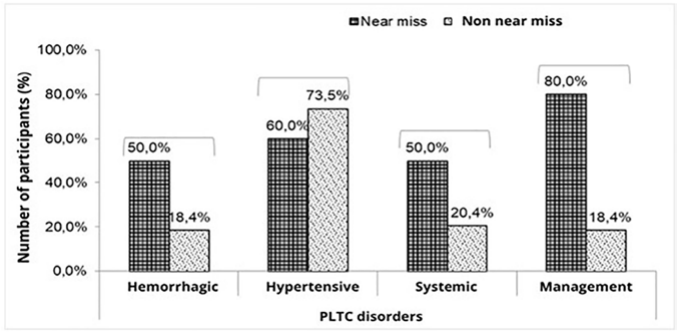
Representation of disorders related to potentially life-threatening conditions of the 59 participants and their associations with the presence of near miss. Campo Grande – MS, 2021.

Note: Values expressed as relative frequency; *Significant association by Fisher’s exact test.

Among the PLTC, hemorrhagic and management disorders were significantly more prevalent among women with near miss (p = 0.04 and p = 0.0003), while hypertensive and systemic disorders did not differ between the groups with or without near miss (p = 0.45 and p = 0.10, respectively) (Figure 1).

Regarding the diagnostic criteria for PLTC, 40 women (67%, n = 40) presented only one diagnostic criterion and 19 (32.2%) presented two or more criteria. Hypertensive disorder was the most prevalent in this group of participants, with a distribution of less than 21.0% among the other disorders analyzed.

Regarding the Near miss women, it was noted that two women presented only one PLTC disorder (20.0%), while the others (80.0%) presented three or more disorders, with management disorders being the most prevalent (80.0%, n = 8), followed by hypertensive disorders (60.0%, n = 6) and, with equal prevalence, hemorrhagic and systemic disorders (50.0%, n = 5).

This indicates a significant association between the number of PLTC and the Near miss classification (p = 0.0001). Regarding the specific criteria for maternal near miss, it was found that three women presented some respiratory dysfunction and another three had hematological or coagulation dysfunctions. It is observed that each of the cardiovascular, neurological and uterine dysfunctions affected two women in this group, with one presenting renal or hepatic dysfunction (Table 4).

**Table 4:** Description of maternal near miss criteria. Campo Grande, MS, 2024.

Maternal near miss criteria	Frequency of observations	
	%	n
Respiratory dysfunction		
Severe tachypnea	2	20.0
Severe hypoxemia	1	10.0
None	7	70.0
Hematologic/coagulation dysfunction		
Large blood or red blood cell transfusion	1	10.0
Severe acute thrombocytopenia	2	20.0
None	7	70.0
Cardiovascular dysfunction		
Shock	2	20.0
None	8	80.0
Neurological dysfunction		
Uncontrollable seizures/status epilepticus	2	20.0
None	8	80.0
Uterine dysfunction		
Uterine bleeding or infection with hysterectomy	2	20.0
None	8	80.0
Kidney Dysfunction		
None	10	100.0
Liver dysfunction		
None		

Note: Values expressed in relative and absolute frequency.

## DISCUSSION

The frequency of maternal near miss in the population studied, over a six-month period was 16% and the prevalence ratio was 9.4 for every 1000 live births. The frequency of maternal near miss can vary from 0.04 to 15% depending on the population studied and the criterion chosen^(13)^.

The reason found is within the variability present in other studies that used the WHO criteria. In a surveillance study carried out in public hospitals in a Brazilian state, the ratio found was 5.4 for every 1000 LB. Another investigation demonstrated a ratio of 8.5 for every 1000 LB. In contrast, a cohort study conducted in a high-risk public maternity hospital in Brazil found a prevalence of 54.8 per 1000 LB. The variation found can be attributed to the difference in population profile, as well as in structure, supplies and hospital support^(10,14,15)^.

Diagnostic criteria based on the WHO are good markers for identifying maternal near miss because they detect severe cases with a higher risk of death, and are considered the gold standard for classifying maternal near miss. However, the importance of making the WHO criteria more flexible and adapting them to regional demand emerges^(16,17)^.

Sociodemographic variables such as skin color, education, and age were not associated with the near miss event, but it is important to highlight that women classified as having potentially life-threatening conditions are mostly non-white, of childbearing age, and with low education. Studies corroborate this finding, showing that such factors are decisive for complications during pregnancy^(18)^.

In a prospective cohort study conducted in Suriname, it was shown that non-white women have twice the chance of developing maternal near miss^(15)^. Another prospective study carried out in Brazil showed that 78% of pregnant women with maternal near miss were non-white and had low-income. No significant association was found with near miss events due to the homogeneity of the profile studied in high-risk maternity hospitals, similar to this study. However, it was emphasized that the ethnicity variable should not be discussed only as a biological factor, but as a social construction directly linked to the environment, and deserves attention^(14)^.

The only sociodemographic variable associated with the occurrence of maternal near miss (NM) was the municipality of residence, which corroborates the results of other studies regarding the influence of the homogeneity of characteristics and the profile of patients treated at the health service studied^(14,18)^.

However, approximately 50% of near miss cases were from regions in inland cities of the state, demonstrating the lack of reference maternal health services in these locations. In a mixed Brazilian study on the verbalization of the experience of postpartum women transferred to Reference Services in Obstetric Emergencies due to maternal near miss, the authors highlighted the main problems as care issues such as non-compliance with protocols and ineffective and violent communication, as well as structural aspects, highlighting flaws in the regionalization, reference, and counter-reference processes^(19)^.

Cases of complications arising from the pregnancy-puerperal cycle force these women to travel to the capital city to receive the necessary care, contributing to a greater risk of poor puerperal outcomes. Women who live in remote areas travel a greater distance to access health services, which can increase the chance of complications and mortality rates^(11)^.

The completion of prenatal care, number of consultations and early initiation of prenatal care did not show a statistical association with the near miss event in this study. It is worth noting that the central-west region, together with the south and southeast, had a higher prevalence of early initiation of prenatal care compared to other Brazilian regions; however, the maternal mortality rate remains high in Brazil, even with the improvement in prenatal coverage in recent years^(20)^.

Other Brazilian studies confirm that women who had complications during pregnancy received prenatal coverage starting early, up to 12 weeks of pregnancy, and an adequate number of consultations, at least six consultations; however, when stricter criteria related to the adequacy of prenatal care are applied, the number drops by almost 70%. These data prove the improvement in prenatal coverage, but question the quality of prenatal care^(21)^.

The classification of pregnancy risk at admission showed that half of the women who experienced near miss received a red or orange classification, which corresponds to emergency care and the need for immediate assistance, while the other half received a green or yellow classification. This association reinforces the near miss indicator, which is also a tool for assessing the in-hospital management received by patients with potentially life-threatening conditions, as well as the use of key interventions and correct management of complications in the pregnancy-puerperal cycle^(7)^.

In many cases, women arrive at the health service in a stable condition, but with obstetric complaints suggestive of complications during pregnancy or the postpartum period. The severity of the woman’s condition associated with an unfavorable maternal outcome may lead, during the hospitalization period, to a failure to use key preventive interventions. In view of this, an articulated maternal care network is required, which provides efficient support in tertiary care^(16)^.

It is important to emphasize that reception with obstetric risk classification is an important support tool in identifying serious cases and making decisions according to the degree of severity, based on scientific evidence^(22)^. The process of embracement with risk classification is of interdisciplinary nature and involves several professionals with different responsibilities, but only the Nurse and the Obstetric Nurse classify the gestational risk^(23)^. Therefore, it is of utmost importance that these professionals have the necessary preparation and scientific basis to support critical judgment and correct decision-making.

Regarding hospital admission, most of the women participating in this study sought health care spontaneously. Of the women who had a near miss, 60% were referred from a hospital with no available beds, or from state inland cities, or from another hospital in the capital. According to resolution number 2077/14 of the Federal Council of Medicine, zero vacancies (*vaga zero*) are an essential resource that guarantees immediate access to patients at risk of death or intense suffering, and should be considered an exceptional situation and not a daily practice in emergency care^(24)^.

This fact demonstrates the frequency of overcrowding in a reference hospital in the state of Mato Grosso do Sul. Conversely, 40% of Near Miss patients sought health services spontaneously, which may be related to the time it took to make the decision to go to the emergency service. Studies confirm the association between decision-making time and travel time to the hospital as predictors of maternal near miss^(25,26)^.

The type of delivery had no significant association with the occurrence of near miss, however 90% of women with near miss and almost 70% of women with PLTC had a cesarean section. In a Brazilian study, the central-west region had the highest rate of cesarean sections in women with gestational complications when compared to other regions^(21)^. It is known that cesarean section is indicated for maternal complications occurring during pregnancy, being considered a highly frequent intervention in Brazil for reasons that are, in most cases, non-clinical, and its occurrence is considered a predictor of maternal near miss^(26,27)^. Furthermore, the risks of maternal morbidities are cyclical since women become pregnant more than once. Women who deliver through cesarean section have an increased risk of placenta previa in a later pregnancy^(27)^.

Hypertensive syndromes were the most frequent clinical complications among women with PLTC without maternal near miss (73.5%) and women with PLTC and maternal near miss (60.0%), homogeneity of data, being the most prevalent cause of serious maternal complications. This information constitutes a marker of a relevant clinical condition, since hypertensive syndromes are the most common obstetric complications found in pregnancy, in addition to being an important cause of death and serious morbidity worldwide^(28)^.

Hypertensive disorders during pregnancy, as one of the predictors of maternal mortality in developing countries, are directly related to sociodemographic factors and access to health care. Therefore, continuous surveillance, use of feasible protocols, risk identification, use of medications, tracking, monitoring, prevention of clinical progression, qualification of professionals and health education are necessary^(26)^.

Bleeding and management disorders are significantly more prevalent among women classified as near miss. This finding also corroborates the ranking of causes that lead to maternal death together with hypertension and puerperal infection^(29)^.

Postpartum hemorrhage and premature placental abruption are among the main causes of maternal mortality, and may be related to the lack of prenatal care, difficulty in accessing information and social factors, as well as the absence of standardized protocols for managing obstetric emergencies^(30)^.

Management criteria were prevalent among women with PLTCs with or without NM. These criteria reveal the use of key interventions in the management of maternal complications with the aim of avoiding tragic maternal outcomes. In other studies, this marker was also shown to be more prevalent when compared to other criteria established by the WHO, and was directly related to a worse prognosis. It is important to note that management markers, such as blood transfusion and return to the operating room, are consequences of a series of unfavorable maternal conditions generally associated with severe pre-eclampsia, eclampsia or hemorrhage^(11,17)^.

The management criteria are part of the WHO classification, which in turn is considered the gold standard in identifying maternal near miss, but studies highlight the need for flexibility depending on the location studied. For example, blood transfusion is considered a management criterion for maternal near miss only when five or more bags are transfused, which may mask the classification of women in serious condition who transfuse less due to the absence or incorrect supply of the blood bank^(17)^.

Furthermore, the high frequency of management criteria indicates that the hospital studied is prepared to handle serious maternal complications, but that improvements are still required due to the absence of a maternal ICU.

The limitations of this study are the reduced collection time and incompleteness of secondary data, in addition to the pandemic period, which impacted the adherence of some women admitted to the service.

However, it is believed that the use of the WHO gold standard as a diagnostic criterion for maternal near miss was valid in bringing the panorama of the theme to the studied scenario, with its results being relevant for healthcare practice, and allowing the method reproduction in other contexts.

## CONCLUSION

Women who had serious complications were characterized as having low education and were self-declared as non-white, data that reaffirm that social determinants influence health and deserve priority in public policy planning. The early start of prenatal care and the number of consultations were satisfactory, but the need to assess the quality of prenatal care is highlighted.

In contrast, living in the countryside, going to the health service, and gestational risk classification were associated with maternal near miss events. Access to health care must be planned and consolidated in the maternal health network, for the specificities of each population. Improving the quality of prenatal care can be decisive in the early diagnosis of gestational complications and potentially influence the decision to seek health services in a timely manner.

In this study, the classification of gestational risk carried out by nurses at the time of hospitalization determined timely intervention. In addition to scientific improvement, these professionals need support in feasible and targeted protocols that reinforce their autonomy in the health field. Specific protocols are suggested with preventive key interventions and notification of NM women, with a view to improving care and consequently reducing the risk of serious maternal outcomes.

## Data Availability

The research data are shared in an open access repository of the Universidade Federal do Mato Grosso do Sul through the link https://repositorio.ufms.br/handle/123456789/4943 as recommended by open science.
