# Supplementary material for: Maternal near miss in a teaching hospital in the Brazilian Midwest: contributions to care
Source: Rev Esc Enferm USP. 2025 Jan 31;58:e20240200. doi: 10.1590/1980-220X-REEUSP-2024-0200en (PMC11785259; doi:10.1590/1980-220X-REEUSP-2024-0200en)
Supplement: Translation

# Translation: Near miss materno em um hospital de ensino do Centro-Oeste brasileiro: contribuições para assistência

**DOI:** 10.1590/1980-220X-REEUSP-2024-0200pt

**Authors:** Aline Amorim da Silveira, Ana Paula de Assis Sales, Andreia Insabralde de Queiroz Cardoso, Elen Ferraz Teston, Adriane Pires Batiston, Renata Marien Knupp Medeiros

**Affiliations:** 1 Universidade Federal do Mato Grosso do Sul, Hospital Maria Aparecida Pedrossian, Campo Grande, MS, Brasil.; 2 Universidade Federal de Mato Grosso do Sul, Instituto Integrado de Saúde, Campo Grande, MS, Brazil.; 3 Universidade Federal de Rondonópolis, Faculdade de Ciências da Saúde, Rondonópolis, MT, Brasil.

**Keywords:** Near Miss, Mortalidade materna, Morbidade, Complicações na gravidez, Enfermagem

## Abstract

**Objetivo:**

Analisar casos de near miss materno em um hospital de ensino

**Método:**

Trata-se de um estudo transversal de abordagem quantitativa. A pesquisa foi realizada em um hospital de ensino, no Mato Grosso do Sul, no período de junho a dezembro de 2021. Os dados foram coletados em prontuários físicos, eletrônicos, exames laboratoriais e caderneta pré-natal, com instrumento estruturado. Posteriormente foram submetidos à análise descritiva e inferencial no Epi Info 7.2.2.6.

**Resultados:**

Foram analisados 59 prontuários, 49 apresentando condição potencialmente ameaçadora da vida e dez *near misses* maternos. Ser procedente do interior do estado, classificação de risco gestacional, desordens hemorrágicas e de manejo apresentaram associações significativas com o near miss materno.

**Conclusão:**

Torna-se necessário o planejamento de ações de rastreio, como, por exemplo, a implantação de um fluxo de identificação de mulheres com condições potencialmente ameaçadoras da vida, e a implementação de protocolos específicos com intervenções-chave preventivas e notificação de near miss materno, tendo em vista aprimoramento do cuidado e consequentemente diminuição do risco de desfecho materno grave.

## INTRODUÇÂO

A redução da mortalidade materna ainda se configura como um desafio em todo o mundo. A terceira meta dos objetivos do desenvolvimento sustentável (ODS), lançada em 2015, propôs a redução da taxa de mortalidade materna global para pelo menos 70 para cada 100.000 nascidos vivos até o ano de 2030^(1)^.

Entretanto, o relatório de tendências de mortalidade materna referente aos primeiros cinco anos de ODS (2016–2020) indicou estagnação no avanço desse objetivo. Em 2020, estima-se que 287 mil mulheres morreram no mundo todo, um terço a menos que o relatado de 2000 a 2015, porém a taxa média anual de redução global caiu de 2,7% para menos de 0,04% nos primeiros cinco anos de ODS^(2)^.

No Brasil, entre 2017 e 2018, todas as regiões do país tiveram queda da razão de mortalidade materna (RMM), com exceção da região Centro-Oeste, que apresentou aumento significativo de 14%, e o estado do Mato Grosso do Sul, com 53,7%. Dentre as principais causas de morte obstétrica no Brasil, destacam-se as doenças hipertensivas, doenças hemorrágicas e a infecção puerperal. No estado de Mato Grosso do Sul, no período de 2017 a 2019, somente a hipertensão teve aumento de 21%^(3)^.

A mortalidade materna é definida como morte de uma pessoa grávida, dentro de 42 dias após a gravidez por qualquer causa relacionada ou agravada pela gravidez, ou seu manejo, mas não por causas acidentais; sendo importante indicador de saúde pública que reflete não só o estado de saúde das mulheres, mas a qualidade dos serviços de saúde^(4,5)^.

Assim como a mortalidade materna, a morbidade materna grave é utilizada como indicador de avaliação da qualidade da assistência obstétrica. A OMS define, hoje, morbidade materna grave como qualquer condição de saúde atribuída ou relacionada a gestação e parto que tenham impacto negativo no bem-estar da mulher^(6)^. Dentro dessa temática, sendo considerado trágico desfecho puerperal, assim como o óbito, emerge o Near miss materno (MNN), definido pela OMS como mulher que quase morreu, mas sobreviveu às complicações ocorridas durante gravidez, parto ou até 42 dias após o término da gravidez^(7)^.

Seus critérios diagnósticos foram padronizados em 2009 por um grupo de trabalho especializado da OMS, que, após análise de diferentes conceitos pré-existentes na literatura, elencaram um conjunto de disfunções orgõnicas capazes de levar uma parturiente ou puérpera a óbito. Foram elencados 25 critérios, divididos em três grupos: clínico, laboratorial e de manejo. Dentro desse contexto, também foi criada uma lista de condições potencialmente ameaçadoras da vida (CPAV) que estão associadas a essas disfunções, incluindo hemorragia pós-parto grave, pré-eclõmpsia grave, eclõmpsia, sepse/infecção e infecção sistêmica grave^(8)^.

O declínio mundial na taxa de mortalidade materna, especialmente em países em desenvolvimento, tornou este indicador uma estratégia menos eficaz para avaliar a qualidade da assistência obstétrica prestada nas instituições de saúde, de forma que a análise do near miss materno pode trazer informações mais robustas nesse sentido. A incorporação da morbidade materna grave e near miss materno é recomendada nas investigações relacionadas a saúde materna^(8,9)^.

Os critérios diagnósticos de near miss materno auxiliam no monitoramento da qualidade de assistência à saúde materna e de vigilõncia de morbidade materna grave. Isso porque a realização de auditoria baseada em casos de near miss materno permite identificar o tipo de assistência recebida nas complicações maternas graves. Esse dado pode viabilizar a melhoria do cuidado, prevenção do agravo de morbidades maternas graves e consequentemente do óbito^(10)^. Além do que, o uso dessa classificação padroniza a metodologia para a identificação de casos graves, a comparação da assistência para gestantes e puérperas ao longo do tempo independentemente da localidade pesquisada^(7,10)^.

Por se tratar de um tema ainda em consolidação nos serviços de saúde, levantamentos amplos e padronizados sobre o NMM são pouco frequentes. Estudos existentes demonstram variação nas taxas de NMM dependendo do critério escolhido e da população estudada tanto em aspectos de estrutura dos serviços de saúde como em aspectos sociais. Estudo realizado em 2022 demonstrou prevalência de NMM com variação mínima de dois por mil nascidos vivos na Itália e máxima de 37,9 por mil nascidos vivos na Etiópia^(11)^.

Em protocolo publicado, de um novo estudo brasileiro em andamento, com período de avaliação contemplando 2021 e 2023, pretende-se estimar a incidência de near miss materno com detecção de amplo número de casos. Porém, como fator limitante, os dados encontrados não poderão ser validados em hospitais com menos de 2750 partos/ano, ressaltando a importõncia de conhecer o perfil da morbidade materna grave em diferentes localidades com serviços de diferentes características como hospitais de menor porte^(5)^.

A incidência e o perfil de casos de morbidade materna grave podem ter comportamentos epidemiológicos distintos. Entretanto, a diferença de dados e de perfis só aprimora as discussões do tema e valida as evidências. Nesse sentido, o presente estudo tem como objetivo identificar e analisar os casos de near miss materno em um hospital de Ensino da região centro-oeste do Brasil.

## MÉTODO

### Tipo de Estudo

Estudo transversal, de abordagem quantitativa, que utilizou os critérios do Strengthening *the Reporting of Observational Studies in Epidemiology* (STROBE) para sua construção^(12)^.

### Local

Foi realizado na maternidade de um hospital de ensino federal de referência, localizado na região centro-oeste brasileira. A maternidade assiste gestantes e puérperas com demanda espontõnea 24 horas/dia e encaminhadas via sistema de regulação, sendo referência em gestação de alto risco e doenças infectocontagiosas, com média 155 partos/mês e 35% de alto risco.

### População e Critérios de Seleção

Foram incluídas no estudo gestantes e puérperas até 42 dias pós-parto, independentemente da idade, que apresentaram pelo menos um critério de condição potencialmente ameaçadora da vida (CPAV), sendo estes caracterizados pela desordem hemorrágica (placenta prévia, placenta acreta/increta/percreta, gestação ectópica, hemorragia pós-parto e rotura uterina), desordem hipertensiva (pré-eclõmpsia grave, eclõmpsia, hipertensão grave, encefalopatia hipertensiva e síndrome HELLP), outras desordens sistêmicas (endometrite, edema pulmonar, falência respiratória, convulsões, sepse, choque, trombocitopenia <100.000 e crise tireotóxica) e indicadores de manejo (transfusão sanguínea, acesso venoso central, histerectomia, admissão em UTI, internação prolongada acima de sete dias, intubação não anestésica, retorno à sala cirúrgica e intervenção cirúrgica).

Foram excluídos do estudo mulheres que não falavam o idioma português, apresentaram incapacidade de resposta ou de compreensão das questões e do TCLE (Termo de consentimento livre e esclarecido) e/ou TALE (Termo de assentimento livre e esclarecido) para menores de 18 anos e que os prontuários não foram encontrados ou que os dados eram insuficientes para responder o objetivo da pesquisa.

### Definição da Amostra

Realizou-se cálculo amostral dos casos de near miss baseado no número esperado de mulheres elegíveis e com desfecho materno grave (variação) conforme o número total de partos^(6)^.

Considerando a prevalência da OMS de 3,7% e que a média de partos anual de 2015 a 2019 no Hospital Universitário Maria Aparecida Pedrossian (HUMAP/UFMS) foi de 1800, a amostra calculada foi de 60 mulheres, com acréscimo de 10% de já possíveis perdas, com 5% de margem de erro e 95% de nível de confiança.

### Coleta de Dados

Os dados foram coletados no período de junho a dezembro de 2021 com auxílio de instrumento elaborado pelo pesquisador principal contendo as variáveis sociodemográficas, dados da assistência, histórico obstétrico, desfecho puerperal e neonatal com ênfase aos critérios de CPAV e NMM. Foi realizado um teste piloto com posterior discussão com os demais pesquisadores para aprovação da versão final do instrumento. Dados de prontuários físicos e eletrônicos de cada paciente, exames e caderneta pré-natal compuseram documentos de análise.

Foi realizada visita diária ao hospital, intercalando entre os períodos da manhã, tarde e noite. Após verificação do censo de internação do centro obstétrico e observados diagnósticos compatíveis com CPAV ou NMM, as mulheres, gestantes ou puérperas foram selecionadas e convidadas a participar do estudo.

Após receber orientações sobre o objetivo do estudo e aceitarem participar, e frente à autorização do TCLE/TALE, os pesquisadores acessavam o prontuário físico, a carteira de prénatal e o sistema AGHUX (Aplicativo de Gestão para hospitais universitários), sistema de gestão e processamento de dados da Rede Ebserh (Emprese brasileira de serviços hospitalares). Posterior a esta coleta dos documentos institucionais, realizava-se a entrevista na enfermaria ou em sala reservada do próprio setor de acordo com a disponibilidade da mulher e do ambiente.

Todas as entrevistas e dados foram coletados uma única vez e em ambiente hospitalar, considerando mulheres internadas por CPAV e Near miss em acompanhamento clínico ou aquelas com o desfecho do parto e no período puerperal, com condição clínica compatível com a participação.

### Tratamento de Dados

Os dados foram analisados no *software* estatístico Epi Info 7.2.2.6., por meio de testes de associações entre duas variáveis categóricas, teste Exato de Fisher e entre mais de duas variáveis pelo Teste Qui quadrado, aplicado um nível de significõncia de 5%.

### Aspectos Éticos

O projeto foi aprovado pelo Comitê de Ética em Pesquisa envolvendo seres humanos, parecer 4.700.375, e seguiu todos os preceitos da resolução n.° 466, de 12 de dezembro de 2012.

## RESULTADOS

Observou-se, em um período de seis meses, a ocorrência de 10 eventos de NMM e frequência de 16% sobre a população elegível. Houve 1062 nascimentos durante o período estudado, configurando uma razão de prevalência de 9,4 para cada 1000 nascidos vivos. Foram incluídas no estudo 59 mulheres, com CPAV, dentre elas 78% de cor não branca e 59,3% com escolaridade fundamental. A média de idade foi de 26,44 ± 6,64 anos (mínimo de 13 e máximo de 41 anos). Houve uma perda por dados incompletos e impossibilidade de complemento através de entrevista. O evento *Near miss* apresentou associação estatística significativa com a procedência de cidades do interior do estado (p = 0,01) conforme se observa na Tabela 1.

**Tabela 1:** Descrição das variáveis sociodemográficas das 59 participantes e suas associações com presença de near miss. Campo Grande – MS, 2021.

Variáveis sócio demográficas	Total	Near miss		Valor de p^(a)^
	% (n)	Sim (10)	Não (49)	
Faixa etária				
≤ 15	3,4 (2)	0,0 (0)	4,1 (2)	0,41
16 a 34	79,7 (47)	70,0 (7)	81,6 (40)	
≥ 35	16,9 (10)	30,0 (3)	14,3 (7)	
Raça/cor				
Branca	22,0 (13)	20,0 (2)	22,4 (11)	1,00
Não branca	78,0 (46)	80,0 (8)	77,6 (38)	
Escolaridade				
Fundamental incompleto/completo	59,3 (19)	60,0 (6)	59,2 (29)	0,66
Médio completo	23,7 (14)	20,0 (2)	24,5 (12)	
Superior completo	6,8 (4)	0,0 (0)	8,2 (4)	
Não informado^(b)^	10,2 (6)	20,0 (2)	8,2 (4)	
Município de Residência				
Capital	81,4 (48)	50,0 (5)	87,8 (43)	0,01*
Interior	18,6 (11)	50,0 (5)	12,2 (6)	

Nota:

^(a)^Análises entre duas variáveis foram realizadas pelo teste Exato de Fisher, enquanto entre mais de duas foram realizadas pelo teste qui quadrado.

^(b)^Não informados = correspondem aos dados ausentes no prontuário cujo montante foi desconsiderado da análise estatística inferencial. *Associação significativa p < 0,05.

Quanto às características obstétricas, foi identificado que a maioria das mulheres eram multíparas e realizaram pré-natal de forma equõnime pré-natal de forma equõnime entre as gestantes posteriormente identificadas com ou sem condição de near miss (p = 0,72 e p = 0,32, respectivamente), sendo que o pré-natal precoce (até a 12^a^ semana gestacional) aconteceu entre a maioria delas.

As características obstétricas estão descritas na Tabela 2. A classificação de risco na internação apresentou associação estatística significativa com condição de near miss (p = 0,005).

**Tabela 2:** Descrição das características obstétricas das 59 participantes e suas associações com presença de near miss. Campo Grande – MS, 2021.

Características obstétricas	Total	Near miss		Valor de p^(a)^
	% (n)	Sim (10)	Não (49)	
Paridade				
Primípara	33,9 (20)	40,0 (4)	32,7 (16)	0,72
Multípara	66,1 (39)	60,0 (6)	67,3 (33)	
Realização do Pré-natal				
Sim	96,6 (57)	90,0 (9)	98,0 (48)	0,32
Não	3,4 (2)	10,0 (1)	2,0 (1)	
Inicio do pré-natal				
Precoce	72,9 (43)	90,0 (9)	69,4 (34)	0,33
Tardio	15,3 (9)	0,0 (0)	18,4 (9)	
Não informado^(b)^	11,9 (7)	10,0 (1)	12,2 (6)	
Número de consultas prénatais				
< 6	30,5 (18)	30,0 (3)	30,6 (15)	1,00
≥ 6	69,5 (41)	70,0 (7)	69,4 (34)	
Risco gestacional pré-natal				
Habitual	47,5 (28)	40,0 (4)	49,0 (24)	1,29
Alto	47,5 (28)	40,0 (4)	49,0 (24)	
Não informado^(b)^	5,1 (3)	20,0 (2)	2,0 (1)	
Classificação de risco na internação				
Verde/Amarelo	84,7 (50)	50,0 (5)	91,8 (45)	0,005*
Laranja/Vermelho	15,3 (9)	50,0 (5)	8,2 (4)	
Doenças maternas pré-existentes				
Prévias	23,7 (14)	30,0 (3)	22,4 (11)	0,49
Gestacionais	33,9 (20)	40,0 (4)	32,7 (16)	
Sem doenças pré-existentes	42,4 (25)	30,0 (3)	44,9 (22)	
Tipo de admissão hospitalar				
Demanda espontõnea	45,8 (27)	40,0 (4)	46,9 (23)	0,005*
Encaminhamento	30,5 (18)	0,0 (0)	36,7 (18)	
Vaga zero	23,7 (14)	60,0 (6)	16,3 (8)	
Período da admissão hospitalar				
1° ou 2° Trimestre gestacional	13,6 (8)	20,0 (2)	12,2 (6)	0,78
3° Trimestre gestacional	67,8 (40)	60,0 (6)	69,4 (34)	
Puerpério imediato (1° ao 10° dia)	18,6 (11)	20,0 (2)	18,4 (9)	

Nota: ^(a)^Análises entre duas variáveis foram realizadas pelo teste Exato de Fisher, enquanto entre mais de duas foram realizadas pelo teste qui quadrado.

^(b)^Não informados = correspondem aos dados ausentes no prontuário cujo montante foi desconsiderado da análise estatística inferencial. *Associação significativa p < 0,05.

Já a admissão por meio de encaminhamento apresentou associação com às não near miss (p = 0,0005). Os dados estão detalhados na Tabela 2.

Quanto aos dados da assistência obstétrica, a cesariana foi a via de nascimento mais utilizada, com maior utilização de medicamentos anti-hipertensivos, ambos sem associação com near miss (p = 0,10 e p = 0,65, respectivamente), conforme se observa na Tabela 3.

**Tabela 3:** Descrição das características da assistência das 59 participantes e suas associações com presença de near miss. Campo Grande – MS, 2021.

Características da assistência	Total	Near miss		Valor de p^(a)^
	% (n)	Sim (10)	Não (49)	
Tipo de parto				
Vaginal	20,3 (12)	0,0 (0)	24,5 (12)	0,10
Cesária	69,5 (41)	90,0 (9)	65,3 (32)	
Não informado^(b)^	10,2 (6)	10,0 (1)	10,2 (5)	
Medicamentos				
Anti-hipertensivo	59,3 (35)	50,0 (5)	61,2 (30)	0,65
Anti-hemorrágico	28,8 (17)	30,0 (3)	28,6 (14)	
Outros	11,9 (7)	20,0 (2)	10,2 (5)	
Local de internação da mulher				
Alojamento conjunto	52,5 (31)	40,0 (4)	55,1 (27)	0,49
CO/CI^(c)^	47,5 (28)	60,0 (6)	44,9 (22)	

Nota: ^(a)^Análises entre duas variáveis foram realizadas pelo teste Exato de Fisher.

^(b)^Não informados= correspondem aos dados ausentes no prontuário cujo montante foi desconsiderado da análise estatística inferencial.

^(c)^CO/CI = Centro obstétrico/Cuidados intermediários ou intensivos.

Dentre as CPAV, as desordens hemorrágicas e de manejo foram significativamente mais prevalentes entre as mulheres near miss (p = 0,04 e p = 0,0003), enquanto as desordens hipertensivas e sistêmicas não diferiram entre os grupos com ou sem near miss (p = 0,45 e p = 0,10, respectivamente) (Figura 1).

**Figura 1:** Representação das desordens relacionadas às condições potencialmente ameaçadoras da vida das 59 participantes e suas associações com presença de near miss. Campo Grande – MS, 2021.

Nota: Valores expressos em frequência relativa; *Associação significativa pelo teste Exato de Fisher.

Quanto aos critérios diagnósticos de CPAV, 40 mulheres (67%, n = 40) apresentaram apenas um critério diagnóstico e 19 (32,2%) apresentaram dois ou mais critérios. A desordem hipertensiva foi a mais prevalente neste grupo de participantes, com distribuição inferior a 21,0% entre as demais desordens analisadas.

Quanto às mulheres Near miss, notou-se que duas mulheres apresentaram apenas uma desordem CPAV (20,0%), sendo que as demais (80,0%) apresentaram três ou mais desordens, sendo as desordens de manejo as mais prevalentes (80,0%, n = 8), seguidas das desordens hipertensivas (60,0%, n = 6) e, com igual prevalência, as desordens hemorrágicas e sistêmicas (50, 0%, n = 5).

Isso aponta uma significativa associação entre o número de CPAV e a classificação Near miss (p = 0,0001). Quanto aos critérios específicos de near miss materno, foi identificado que três mulheres apresentaram alguma disfunção respiratória e, outras três, disfunções hematológicas ou de coagulação. Observa-se que cada uma das disfunções cardiovascular, neurológica e uterina acometeram duas mulheres deste grupo, sendo que se observou disfunção renal ou hepática (Tabela 4).

**Tabela 4:** Descrição dos critérios de near miss materno. Campo Grande – MS, 2021 (n = 10).

Critérios de near miss materno	Frequência de observações	
	%	n
Disfunção respiratória		
Taquipneia grave	2	20,0
Hipoxemia grave	1	10,0
Nenhum	7	70,0
Disfunção hematológica/coagulação		
Grande transfusão de sangue ou de hemácias	1	10,0
Trombocitopenia aguda grave	2	20,0
Nenhum	7	70,0
Disfunção cardiovascular		
Choque	2	20,0
Nenhum	8	80,0
Disfunção neurológica		
Convulsões incontroláveis/status epilético	2	20,0
Nenhum	8	80,0
Disfunção uterina		
Hemorragia ou infecção uterina com histerectomia	2	20,0
Nenhum	8	80,0
Disfunção Renal		
Nenhum	10	100,0
Disfunção hepática		
Nenhum		

Nota: Valores expressos em frequência relativa e absoluta.

## DISCUSSÃO

A frequência de near miss materno sobre a população estudada, em um período de seis meses, foi de 16%, e a razão de prevalência foi de 9,4 para cada 1000 nascidos vivos. A frequência de near miss materno pode variar de 0,04 a 15% dependendo da população estudada e o critério escolhido^(13)^.

A razão encontrada está dentro da variabilidade presente em outras pesquisas que utilizaram os critérios da OMS. Em estudo de vigilõncia realizado em hospitais públicos de um estado brasileiro, a razão encontrada foi de 5,4 para cada 1000 NV. Outra investigação demonstrou uma razão de 8,5 para cada 1000 NV. Em contrapartida, uma coorte realizada em uma maternidade pública de alto risco do Brasil encontrou uma prevalência de 54,8 para cada 1000 NV. A variação encontrada pode ser atribuída à diferença de perfil da população, bem como de estrutura, insumos e suporte hospitalar^(10,14,15)^.

Critérios diagnósticos baseados na OMS são bons marcadores na identificação do near miss materno por detectarem casos graves com maior risco de morte, sendo considerados padrão ouro para classificação de near miss materno; porém, emerge a importõncia de flexibilização dos critérios da OMS com adaptação para demanda regional^(16,17)^.

Variáveis sociodemográficas como cor da pele, escolaridade e idade não foram associadas ao evento near miss, mas é relevante ressaltar que as mulheres classificadas com condições potencialmente ameaçadoras da vida são, em sua maioria, não brancas, em idade fértil e com baixa escolaridade. Estudos corroboram tal achado evidenciando esses fatores como determinantes para complicações na gravidez^(18)^.

Em coorte prospectiva realizada no Suriname, foi evidenciado que mulheres não brancas têm o dobro de chance de desenvolver near miss materno^(15)^. Outro estudo prospectivo realizado no Brasil evidenciou que 78% das gestantes com near miss materno eram não brancas e eram de baixa renda. Não foi encontrada associação significativa com evento near miss pela homogeneidade do perfil estudado em maternidade de alto risco, semelhante a esse estudo; porém, salientou-se que a variável etnia não deve ser discutida somente como fator biológico, mas como construção social que está diretamente ligada ao ambiente e merece atenção^(14)^.

A única variável sociodemográfica associada à ocorrência de Near miss materno (NM) foi o município de residência, o que corrobora resultados de outros estudos no sentido da influência da homogeneidade das características e pelo perfil de pacientes atendidas no serviço de saúde estudado^(14,18)^.

No entanto, aproximadamente 50% dos casos de near miss eram de regiões provenientes do interior do estado, evidenciando a não existência de serviços de saúde materna de referência nestas localidades. Em estudo misto brasileiro, sobre verbalização da vivência de puérperas transferidas para Serviços de Referência em Emergências Obstétricas por near miss materno, os autores evidenciaram como principais problemas as questões assistenciais, como inobservõncia de protocolos e comunicação ineficaz e violenta, bem como aspectos estruturais, evidenciando falhas nos processos de regionalização, referência e contrarreferência^(19)^.

Casos de complicações decorrentes do ciclo gravídico puerperal obrigam o deslocamento dessas mulheres até a capital para receber o atendimento necessário, contribuindo com risco maior de mau desfecho puerperal. Mulheres que residem em áreas afastadas percorrem uma distõncia maior para ter acesso aos serviços de saúde, o que pode aumentar a chance de complicações e taxa de mortalidade^(11)^.

A realização do pré-natal, número de consultas e o início precoce do pré-natal não apresentaram associação estatística com o evento near miss neste estudo. Cabe destacar que a região centro-oeste, juntamente com o sul e sudeste, apresentaram maior prevalência de início precoce do pré-natal em comparação com as demais regiões brasileiras; no entanto, a taxa de mortalidade materna permanece elevada no Brasil, mesmo com a melhora, nos últimos anos, da cobertura pré-natal^(20)^.

Outros estudos brasileiros confirmam que mulheres que tiveram intercorrência na gestação receberam cobertura pré-natal com início precoce, até 12 semanas de gestação, e número adequado de consultas, no mínimo seis consultas, porém quando aplicados critérios mais rígidos relacionados a adequação do pré-natal o número cai quase que 70%. Estes dados comprovam a melhoria da cobertura pré-natal, mas questionam a qualidade do pré-natal^(21)^.

A Classificação de risco gestacional na internação apontou que metade das mulheres que experimentaram near miss receberam classificação vermelha ou laranja, o que condiz com atendimento de emergência e a necessidade de assistência imediata, enquanto a outra metade recebeu classificação verde ou amarela. Essa associação reforça que o indicador near miss também é uma ferramenta de avaliação do manejo intra-hospitalar recebido por pacientes com condições potencialmente ameaçadoras da vida, bem como do uso de intervenções-chave e correto manejo das complicações do ciclo gravídico puerperal^(7)^.

Em muitos casos a mulher chega ao serviço de saúde estável, porém com queixas obstétricas sugestivas de complicações da gestação ou do puerpério. A gravidade do estado da mulher com associação de desfecho materno desfavorável pode causar, durante o período de internação, falha na utilização de intervenções-chave preventivas. Frente a isso, é necessária uma rede de assistência materna articulada, que ofereça suporte eficiente na atenção terciária^(16)^.

É importante ressaltar que o acolhimento com classificação de risco obstétrico é uma importante ferramenta de apoio na identificação de casos graves e tomada de decisão conforme o grau de gravidade, baseada em evidências científicas^(22)^. O processo de acolhimento com classificação de risco tem caráter interdisciplinar e envolve vários profissionais com atribuições distintas, porém somente o Enfermeiro e o Enfermeiro Obstetra classificam o risco gestacional^(23)^. Portanto, é de suma importõncia o preparo e embasamento científico que subsidiem julgamento crítico e tomada de decisões corretas por parte destes profissionais.

Com relação à admissão hospitalar, a maioria das mulheres participantes desse estudo procurou o atendimento de saúde por demanda espontõnea. Das mulheres que tiveram near miss, 60% foram encaminhadas por vaga zero, ou do interior do estado, ou de outro hospital da capital. Segundo a resolução número 2077/14 do Conselho Federal de Medicina, vaga zero é um recurso essencial que garante acesso imediato a pacientes com risco de morte ou sofrimento intenso, devendo ser considerada uma situação de exceção e não uma prática cotidiana na atenção às urgências^(24)^.

Esse fato demonstra a frequência da superlotação em um hospital de referência no estado de Mato Grosso do Sul. Por outro lado, 40% das pacientes Near miss procuraram o serviço de saúde de forma espontõnea, o que pode estar relacionado com o tempo de demora para tomada de decisão de ir até o serviço de urgência. Estudos confirmam que a associação do tempo de tomada de decisão ao tempo de deslocamento até o hospital são preditores de near miss materno^(25,26)^.

O tipo de parto não teve associação significativa com a ocorrência de near miss; contudo, 90% das mulheres com near miss e quase 70% das mulheres com CPAV tiveram parto cesariana. Em estudo brasileiro, a região centro-oeste teve a maior taxa de cesariana em mulheres com intercorrências gestacionais quando comparada com as demais regiões^(21)^. Sabe-se que a cesariana é indicada por complicações maternas ocorridas na gestação, sendo considerada uma intervenção de frequência elevada no Brasil por motivações, na maioria das vezes, não clínicas, e sua ocorrência é considerada preditor para near miss materno^(26,27)^. Ademais, os riscos de morbidades maternas são cíclicos, já que as mulheres ficam grávidas mais de uma vez. Mulheres que dão à luz por cesariana têm risco aumentado de placenta prévia em uma gestação posterior^(27)^.

As síndromes hipertensivas mostraram-se as complicações clínicas mais frequente entre mulheres com CPAV não near miss materno (73,5%) e mulheres com CPAV e near miss materno (60,0%), homogeneidade dos dados, e a causa mais prevalente de complicações maternas grave. Essa informação constitui marcador de condição clínica relevante, visto que as síndromes hipertensivas são as complicações obstétricas mais comuns encontradas na gravidez, além de ser causa importante de morte e morbidade grave em todo o mundo^(28)^.

Os distúrbios hipertensivos na gestação, como um dos preditores para a mortalidade materna em países em desenvolvimento, têm relação direta com os fatores sociodemográficos e acesso à saúde. Assim, faz-se necessária uma vigilõncia contínua, uso de protocolos factíveis, identificação de risco, utilização de medicamentos, rastreamento, monitoramento, prevenção de evolução do quadro clínico, qualificação de profissionais e educação em saúde^(26)^.

As desordens hemorrágicas e de manejo se sobressaem significativamente entre as mulheres classificadas como near miss. Esse achado também corrobora o ranking de causas que levam a morte materna juntamente com hipertensão e infecção puerperal^(29)^.

A hemorragia pós-parto e o descolamento prematuro de placenta encontram-se entre as principais causas de mortalidade materna, e podem estar relacionados ao déficit do pré-natal, dificuldade de acesso, informação e fatores sociais, bem como ausência de protocolos padronizados para manejo de emergências obstétricas^(30)^.

Os critérios de manejo mostraram-se prevalentes entre as mulheres com CPAVs com ou sem NM. Esses critérios revelam o uso de intervenções-chave no manejo de complicações maternas com objetivo de evitar desfecho materno trágico. Em outros estudos, esse marcador também se mostrou mais prevalente se comparado aos demais critérios estabelecidos pela OMS, e teve relação direta com o pior prognóstico. Ressalta-se que os marcadores de manejo, tais como transfusão sanguínea, retorno a sala cirúrgica, são consequências de uma série de condições maternas desfavoráveis geralmente associadas a pré-eclõmpsia grave, eclõmpsia ou hemorragia^(11,17)^.

Os critérios de manejo fazem parte da classificação da OMS que, por sua vez, é considerada padrão ouro na identificação de near miss materno. Porém, estudos ressaltam a necessidade de flexibilização a depender do local estudado, a exemplo de transfusão sanguínea, que é considerada um critério de manejo para near miss materno apenas quando se transfundem cinco bolsas ou mais, podendo mascarar classificação de mulheres em estado grave quando transfundem menos por ausência ou não abastecimento correto do banco de sangue^(17)^.

No mais, a alta frequência dos critérios de manejo denotam preparo de estrutura compatível para atendimento de complicações maternas graves no hospital estudado, mas que ainda carece de aprimoramentos pela ausência de UTI materna.

Considera-se como limitação deste estudo o tempo de coleta reduzido e a incompletude de dados secundários, além do momento pandêmico, fato este que impactou na adesão de algumas mulheres internadas no serviço.

Contudo, acredita-se que a utilização do padrão ouro da OMS como critério diagnóstico de Near miss materno foi válida por trazer o panorama da temática no cenário estudado, sendo seus resultados relevantes para prática assistencial, podendo o método ser reproduzido em outros contextos.

## CONCLUSÂO

As mulheres que sofreram complicações graves foram caracterizadas como tendo baixa escolaridade e sendo autodeclaradas não brancas, dados que reafirmam que os determinantes sociais influenciam na saúde e merecem prioridade no planejamento de políticas públicas. O início precoce do acompanhamento pré-natal e o número de consultas foram satisfatórios, mas salienta-se a necessidade de avaliação da qualidade do pré-natal.

Em contrapartida, residir no interior, deslocamento ao serviço de saúde e classificação de risco gestacional tiveram associação com evento near miss materno. É necessário que o acesso à saúde deva ser planejado e consolidado na rede de saúde materna, de acordo com as especificidades de cada população. A intensificação na qualidade do pré-natal pode ser resolutiva no diagnóstico precoce de complicações gestacionais e potencial influenciador na decisão da procura em tempo oportuno do serviço de saúde.

Nesse estudo a classificação de risco gestacional realizada por enfermeiros, no momento da internação, determinou intervenção em tempo oportuno. Além de aprimoramento científico, é preciso que esses profissionais tenham suporte em protocolos factíveis e direcionados que reforcem sua autonomia no õmbito de saúde. Sugere-se realizar protocolos específicos com intervenções-chave preventivas e notificação de mulheres NM, tendo em vista aprimoramento do cuidado e consequentemente diminuição do risco de desfecho materno grave.

## Disponibilidade de Dados

Os dados da pesquisa estão compartilhados em repositório de acesso aberto da Universidade Federal do Mato Grosso do Sul por meio do link https://repositorio.ufms.br/handle/123456789/4943 conforme preconizado pela ciência aberta.
